# Knotted artifacts in predicted 3D RNA structures

**DOI:** 10.1371/journal.pcbi.1011959

**Published:** 2024-06-20

**Authors:** Bartosz A. Gren, Maciej Antczak, Tomasz Zok, Joanna I. Sulkowska, Marta Szachniuk

**Affiliations:** 1 Centre of New Technologies, University of Warsaw, Warsaw, Poland; 2 Institute of Computing Science, Poznan University of Technology, Poznan, Poland; 3 Institute of Bioorganic Chemistry, Polish Academy of Sciences, Poznan, Poland; University of Missouri, UNITED STATES

## Abstract

Unlike proteins, RNAs deposited in the Protein Data Bank do not contain topological knots. Recently, admittedly, the first trefoil knot and some lasso-type conformations have been found in experimental RNA structures, but these are still exceptional cases. Meanwhile, algorithms predicting 3D RNA models have happened to form knotted structures not so rarely. Interestingly, machine learning-based predictors seem to be more prone to generate knotted RNA folds than traditional methods. A similar situation is observed for the entanglements of structural elements. In this paper, we analyze all models submitted to the CASP15 competition in the 3D RNA structure prediction category. We show what types of topological knots and structure element entanglements appear in the submitted models and highlight what methods are behind the generation of such conformations. We also study the structural aspect of susceptibility to entanglement. We suggest that predictors take care of an evaluation of RNA models to avoid publishing structures with artifacts, such as unusual entanglements, that result from hallucinations of predictive algorithms.

## Author summary

- 3D RNA structure prediction contests such as CASP and RNA-Puzzles lack measures for topology-wise evaluation of predicted models. Thus, predictors happen to submit potentially inappropriate conformations, for example, containing entanglements that are prediction artifacts.
- Automated identification of entanglements in 3D RNA structures is computationally hard. Distinguishing correct from incorrectly entangled conformations is not trivial and often requires expert knowledge.
- We analyzed 3D RNA models submitted to CASP15 and found that all entanglements in these models are artifacts.
- Compared to non-ML, machine learning-based methods are more prone to generating entanglements that are not present in natural RNAs.
- To increase the reliability of 3D RNA structure prediction, it is necessary to reject abnormally entangled structures in the modeling stage.

## Introduction

The birth of the third decade of this century brought a sudden surge of interest in modeling 3D RNA structures. The latter was, among other things, a by-product of the COVID-19 pandemic, whose main actor was the RNA virus, and intensive research on the development of RNA-based vaccines against COVID-19. The second major factor was the spectacular success brought about by the application of deep neural networks to model protein structures [1]. As a result, new methods have emerged to predict RNA structures, most of which use machine learning models in an end-to-end approach or at selected stages of the 3D folding process [2]. Many of them have undergone virgin benchmarking while competing in recent RNA-Puzzles and CASP15 initiatives, which aim to blindly evaluate predicted 3D RNA models and identify the best predictive tools. The results of both competitions show that none of the new methods has made a breakthrough in the quality and accuracy of the prediction of the 3D RNA structure so far. The latter are evaluated using various measures of distance (DI, GDT-TS, lDDT, MCQ, RMSD), similarity (INF, TM-score, LCS), and quality (Clash score) [3–8]. No measure can directly assess the accuracy of the 3D model topology and its compatibility with the topology of the target. Consequently, awareness of topological irregularities in 3D RNA predictions is negligible in the RNA community, and the predicted models happen to contain them. These anomalies include entanglements that are absent from known experimental structures. We can consider them locally, taking into account the secondary and tertiary structure of the molecule (we then speak of entanglements of structural elements), and globally, studying the spatial arrangement of the RNA backbone (we then analyze topological knots).

**Fig 1:**
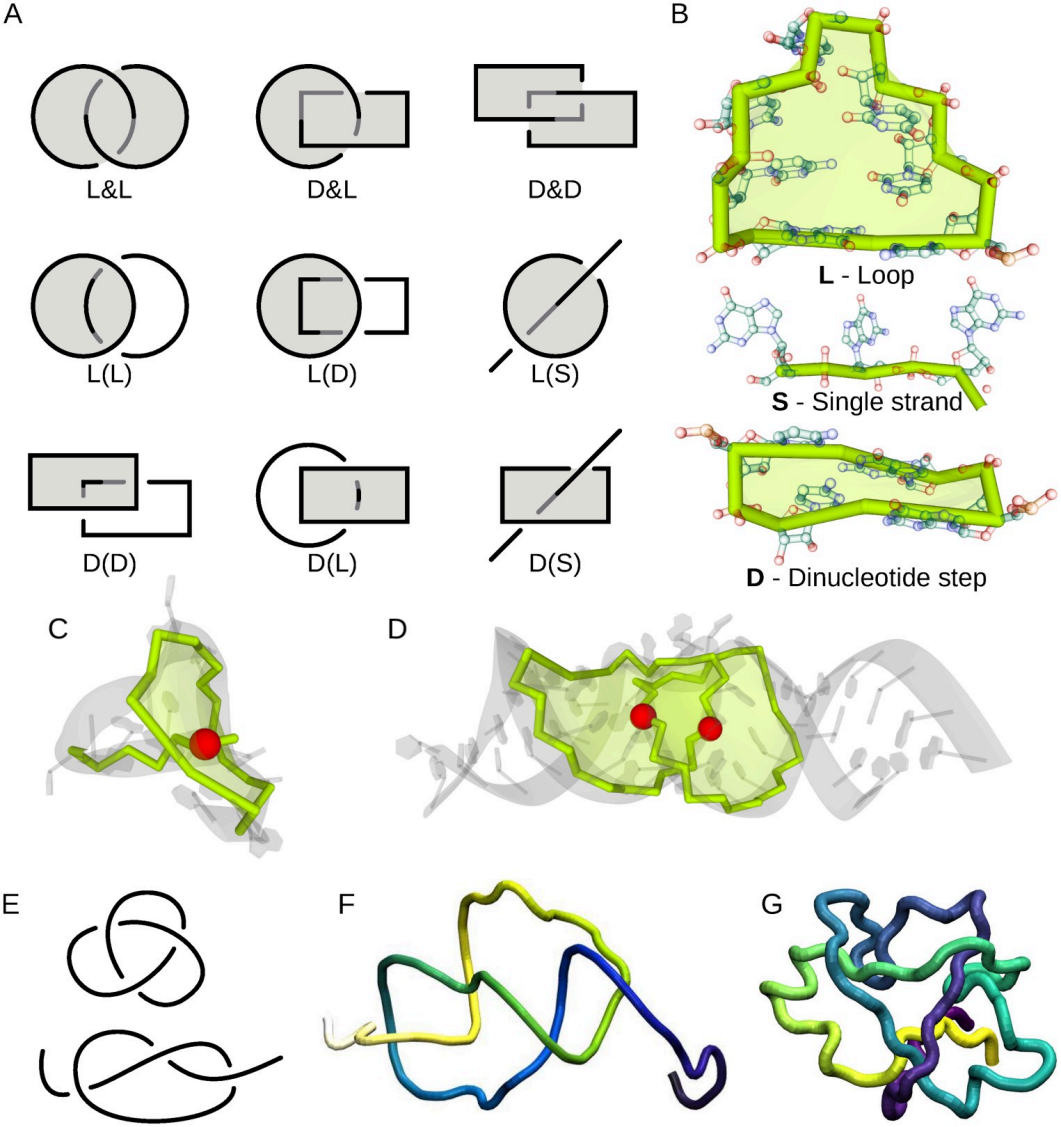
Types of entanglements. (A) Schematic drawings of interlaces (D&D, D&L, L&L) and lassos (D(D), D(L), D(S), L(D), L(L), L(S)). Loop (L) is represented by a circle, dinucleotide step (D) by a rectangle, and single strand (S) by a segment. (B) Example structural elements L, D, and S, and entanglements of structure elements—(C) L(S)-type lasso formed by a 6-nt apical loop and the 5’-end threaded through it, (D) misfolded conformation of two loops forming L&L-type interlace instead of kissing loops. (E) Diagrams of closed and opened trefoil knot, and two molecules with trefoils—(F) sRNA RydC (PDB ID: 4V2S:G [18]) and (G) PHD finger-like domain-containing protein 5A (PDB ID: 5ZYA:C [19]).

The entanglement of structural elements occurs when two elements of the RNA structure are in spatial conflict, that is, one of them punctures the other to form a lasso, interlace (known as a link in knot theory) [9] (Fig 1A) or genus type (the genus trace represents how interconnected and densely packed the structure is in three dimensions) [10] (Fig 1E). Elements such as loops, stems (consisting of dinucleotide steps), and single-stranded fragments can be involved in entanglements of structure elements (Fig 1B). From the viewpoint of knot theory, lassos are not knots. Interlaces can be interpreted as Hopf links (the simplest type of two interlaced loops) defined on loops traced by the phosphodiester and hydrogen bonds; the first contribute to the formation of the nucleotide chain and the latter to base pairs. The formation of many types of entanglements of structure elements, like some interlaces (loop & dinucleotide step, dinucleotide step & dinucleotide step) and deep lassos, contradicts the RNA folding hierarchy and is therefore hardly possible. We do not find them in high-resolution experimentally determined RNAs [11]. They occur in structures modeled *in silico*, in which case they should be regarded as artifacts of computational procedures.

The formation of topological knots in RNA structures has been studied by a few research teams so far. The authors of [12], although they came across knotted 16S rRNA domains, argued about the low likelihood of topological knots in large native RNAs and suggested examining experimental RNA models for knotting before their publication. In this spirit, authors of [13, 14] created a method to assemble viral RNA genomes that avoided the creation of topological defects. Micheletti *et al*. [15] found three knotted rRNAs solved by cryo-EM, but due to the absence of knots in high-resolution structures, concluded on some thermodynamic or kinetic mechanisms that minimize the entanglement of biologically viable structural RNAs. Yet a little later, the same group suggested that the properties of some of the predicted RNA secondary structures indicate the potential to form knots [16]. Only recently, a trefoil (3_1_), the first non-trivial topological knot, was identified in high-resolution experimental RNA structure [17]. However, this is not sufficient to infer the formation of knots in RNA structures and their possible influence on the function of the molecule, as we can for proteins [20–24]. In addition, in the case of proteins, 6 different types of knots (3_1_, 4_1_, 5_2_, 6_1_) have already been identified [25], including those predicted by AI methods such as 3_1_#3_1_ [26] and 7_1_ [27], whose structure has been experimentally confirmed while other more complex knots [28] await confirmation.

Theoretical studies on ideal (ghost) polymers predict that probability of polymer being unknotted *P*_unknot_ falls exponentially with increasing length *L* and the inverse of the Kuhn length *b* of the chain: *P*_unknot_(*L*;*b*) = exp(−*αL*/*b*) [29–31], where the Kuhn length *b* is a quantity proportional to stiffness, and *α* is a constant that depends on a theoretical model used. This behavior is not observed for RNA, as the only known knotted RNA molecule is only 65 nt long [17]. In addition, no correlation was found between the size of the RNA molecule and its susceptibility to form entanglements of structural elements [9]. RNAs are brush-like real biopolymers living in a polar, polyelectrolyte environment, and their folding is driven by the minimization of free energy (theirs and the solvents). Research on the folding of knotted proteins reveals that although the knotted state may enhance protein stability, a topological energy barrier must first be overcome to reach the native knotted state [32–38]. This topological energy barrier hinders the formation of knotted proteins. Since methods that predict the native state of biopolymers do not fold molecules *de novo* but directly guess these native states, they have a harder job avoiding states unavailable due to topological energy barriers [39, 40].

In this work, we have analyzed all 3D RNA models predicted in the recent CASP competition. Using existing computational tools, RNAspider [11] and Topoly [41], we have scanned these predictions for entanglements of structural elements and topological knots. We have studied the structural and methodological aspects of susceptibility to entanglement generation in RNA models. All entanglements found are artifacts of the modeling procedures. Methods using deep learning entangle RNA chains more often than non-ML algorithms and generate quite complex topological knots. We believe that predictive methods should automatically reject models with invalid chain entanglements. This would improve the reliability and quality of the prediction of the 3D RNA structure.

## Materials and methods

### Benchmark data

To identify, count, and classify knots and entanglements of structural elements in the 3D RNA structure models predicted in CASP15, we downloaded the data from the competition website in September 2023. The CASP15 resources are available at https://predictioncenter.org/download_area/CASP15/predictions/RNA/ [42]). Data were cleaned of irrelevant metadata using the clean-casp-headers.awk script and grouped by 12 RNA targets: R1107 (69 nt), R1108 (69 nt), R1116 (157 nt), R1117 (30 nt), R1126 (363 nt), R1128 (238 nt), R1136 (374 nt), R1138 (720 nt), R1149 (124 nt), R1156 (135 nt), R1189 (118 nt) and R1190 (118 nt). The data set contained a total of 62 reference structures (for some targets, there was more than one structure) and 1,660 models computationally predicted by 41 modeling groups.

### Methods used for entanglements of structure elements

The RNAspider web server was applied to identify and classify entanglements of structure elements in 3D RNA structures [11]. The system detects lassos and interlaces and assigns them to nine subclasses (Fig 1A and 1B)—L&L, L&D, D&D, L(S), L(D), L(L), D(S), D(D), and D(L)—based on the types of elements involved, i.e., loops, dinucleotide steps (both are closed structural elements), and single-stranded fragments (open element). It was run with the default settings of the advanced parameters. We then calculated the depth of each identified lasso formed from the loop L(*), where * stands for S, D, or L. This was done using an additional script (2_analyze_depth.py [43]), as RNAspider does not cover such functionality. The script was fed with information on the intersection points that RNAspider provided for every closed structure element. The intersection point is determined by 3D coordinates, where the backbone or hydrogen bond in a canonical base pair punctures a surface that covers a closed element. We considered two cases. In the case of L(S), when a single strand is taken to lasso by the loop, we computed the depth as the minimum number of its nucleotides towards the 5’ and 3’ ends from the intersection point. In the second case, when a closed element is lassoed (L(L), L(D)) or when a single strand punctures the loop twice (L(S.)), the lasso is characterized by two intersection points. Therefore, we computed the depth as the number of nucleotides between the intersection points. Knowing the depth, we divided the lassos created by the loops into shallow (depth ≤ 5 nts) and deep (depth > 5 nts). Following our experience with molecular dynamics, we hypothesize that shallow lassos L(*) can spontaneously disentangle during structure folding and therefore may not be treated as structure anomalies even though no lassos occur in the reference structures. In contrast, we consider interlaces, D(*) lassos, and deep L(*) lassos as modeling artifacts.

### Methods used for topological knots

Topological knots were detected and identified using the Topoly Python package [41]. This collection of scripts offers features to study the topology of polymers and a generation of artificial, random polymer chains of a given topology. Here, the coordinates of the sugar-phosphate backbone atoms (P, O5′, C5′, C4′, C3′, O3′) were extracted from the 3D structure data. To identify knots, the Alexander polynomial [44] was calculated using the topoly.alexander() function, which before calculations closes the chain randomly by projecting RNA endpoints on the big sphere around the molecule and connecting them (two-point probabilistic closure, 200 closures, explained more in [45, 46]). We treated the structure as knotted if <50% of the closures were unknots. At first, Topoly identified 84 knotted RNAs. They were visually inspected to confirm entanglements. For some of them, a direct closure seemed to make more sense. We discarded 7 of these models since they were unknots after direct closure. 67 predictions failed to process by Topoly due to non-unique atom coordinates (47 models) or too tangled structure (20 models). Structures in the latter subset were visually inspected and confirmed to be too densely packed to be correct. We assigned them to a category named TTC (too tangled to check) and did not include them in further analysis, unlike trefoils (Fig 1C) and complex knots with known classification.

## Results and discussion

**Fig 2:**
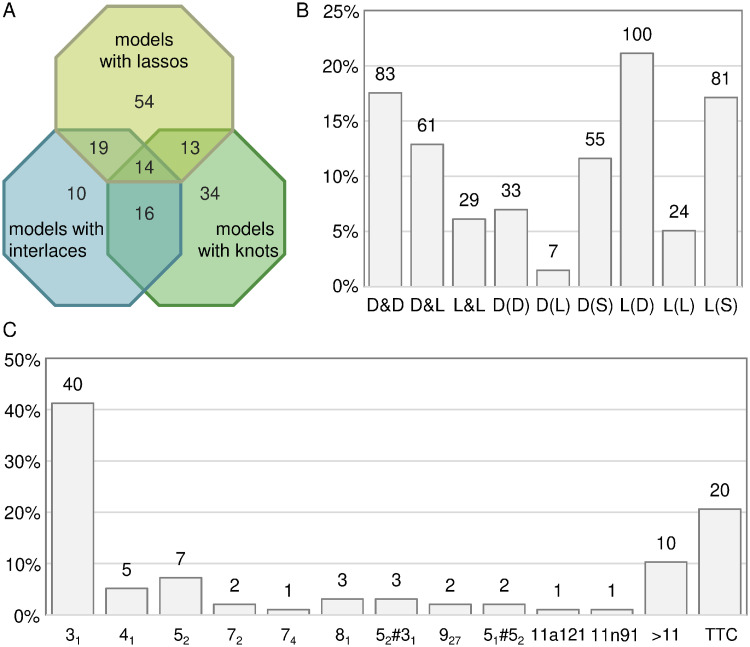
**(A) Models with various entanglement types in numbers. (B) Entanglements of structure elements and (C) topological knots in RNA predictions by type**. Column labels in (B) and (C) show the total number of entanglements of a given type across all predictions.

In this work, we analyzed entanglements in 3D RNA models submitted to CASP15 following the pipeline presented in S1 Fig. We took into account the entanglements of structural elements and topological knots. It is important to emphasize that none of the reference structures, of which there were 62 models, was entangled from the point of view of the structure elements or topological knots. In contrast, of the 1,660 predicted models, 160 models have either entanglements of structure elements or topological knots, 83 models have only entanglements of structural elements, 34 models have only topological knots, and 43 models have both (S1 Table). Fig 2A shows a Venn diagram detailing the number of predicted models that contain given types of entanglements. Note that the existence of topological knots in the 3D RNA model does not equate to entanglement of the structural elements of the model and vice versa. In 126 entangled models, we encountered 173 interlaces and 300 lassos (Fig 2B). The latter group included 67 deep and 33 shallow conformations of the L(*) type. Note that among the lassos, the L(S) and L(D) types are most abundantly represented in the RNA predictions. Intuition dictates that this is entirely reasonable since forming a lasso around a single or double helix should be relatively easy. In contrast, pushing a non-single-stranded fragment of a structure through a dinucleotide step or a loop through a loop seems quite difficult. Therefore, these types of entanglements are relatively rare in the models. In 77 RNA models with topological knots, we found a predominance of trefoils—they account for more than half of all knots with assigned classes excluding the TTC subset (Fig 2C). TTC, the second large subgroup, is made up of 20 models whose knotting is too complex to classify correctly.

### Target-focused analysis

**Fig 3:**
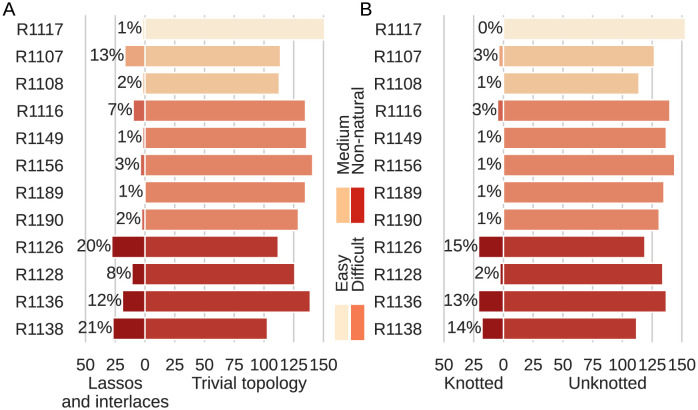
Distribution of entangled 3D RNA structure predictions by target. Target structures are grouped by difficulty [8]. Results are displayed separately for (A) entanglements of structure elements (lassos and interlaces) and (B) topological knots.

In this part of our experiment, we focused on the structural aspect of the structure entanglement problem. We asked whether, among the RNA targets in CASP15, we could distinguish structures that show a greater /lower susceptibility to entanglement during computer modeling. Answering this question required analyzing the predicted models clustered by target (see Fig 3 and Table 1).

**Table 1:** Entanglements in RNA predictions by target.

Predictions for	#Models	#Interlaces	#Lassos	#Trefoils	#Other knots	#TTC
natural targets	1,095	17	62	14	0	0
synthetic targets	565	156	232	26	37	20
total number	1,660	173	294	40	37	20

The target structures were divided by the CASP assessors into natural (8 targets) and synthetic (4 targets). The former cluster distinguished between easy (R1117), medium (R1107, R1108), and difficult (R1116, R1149, R1156, R1189, R1190) targets. Non-natural ones included R1126, R1128, R1136, and R1138. Assignment of a target to particular group indicates the ease/difficulty of its structure prediction due to the similarity to known experimental structures [8]. In general, 33% (41) of the entangled structures are predictions of natural RNA targets, and 67% (85) are models from the non-natural cluster.

Based on the analysis of entanglements of structural elements (Fig 3A), we can say that the probability of entangled predictions for natural RNAs equals 0.03, while for non-natural targets it is 0.15, which is 5x higher. If we look at the sets of predictions per target, we can see that the entangled structures represent 8–20% for the synthetic targets. In contrast, in the sets of models for natural targets, the percentage of entangled predictions is 0.74–2.76%. The exceptions here are R1107 (12.98%) and R1116 (6.90%), the former of which is a moderately difficult structure with a pseudoknot, and the latter is classified as difficult.

The diagram prepared for the topological knots (Fig 3B) has similar characteristics as in the case of entangled structure elements. The highest number of knotted models is found for the targets for which we observe the most entanglements of structural elements. The study reveals that the knotting probability is 0.01 for natural structures and 0.12 (10x higher) for non-natural ones. All knotted predictions of natural structures are trefoils (3_1_, simplest non-trivial knot). For non-natural structures, only more complex knots appear, moreover, they make up the majority of knotted structures.

Some predictions contain more than one entanglement. Table 1 presents a distribution of various types of entanglements across predictions for natural and synthetic targets. For simplicity, topological knots were divided into trefoils (the simplest non-trivial knot) and other knots (more complicated ones). The rightmost column represents unclassified knots from non-physically dense structures (TTC—too tangled to check knot type).

Finally, let us add that the largest number of entangled 3D models (both from the point of view of topological knots and entangled structure elements) was identified in the predictions for the three largest targets, R1138 (720 nts), R1136 (374 nts), and R1126 (363 nts), all of which are synthetic. However, note that, as shown in [9], no simple relationship has yet been observed between the entanglements of structural elements and the size of the structure. Given that topological knots more complex than trefoil are found only in the predictions of synthetic targets, it seems that the large number of entanglements and their complexity are a result of the specificity of the non-natural target rather than the structure size.

### Method-focused analysis

**Fig 4:**
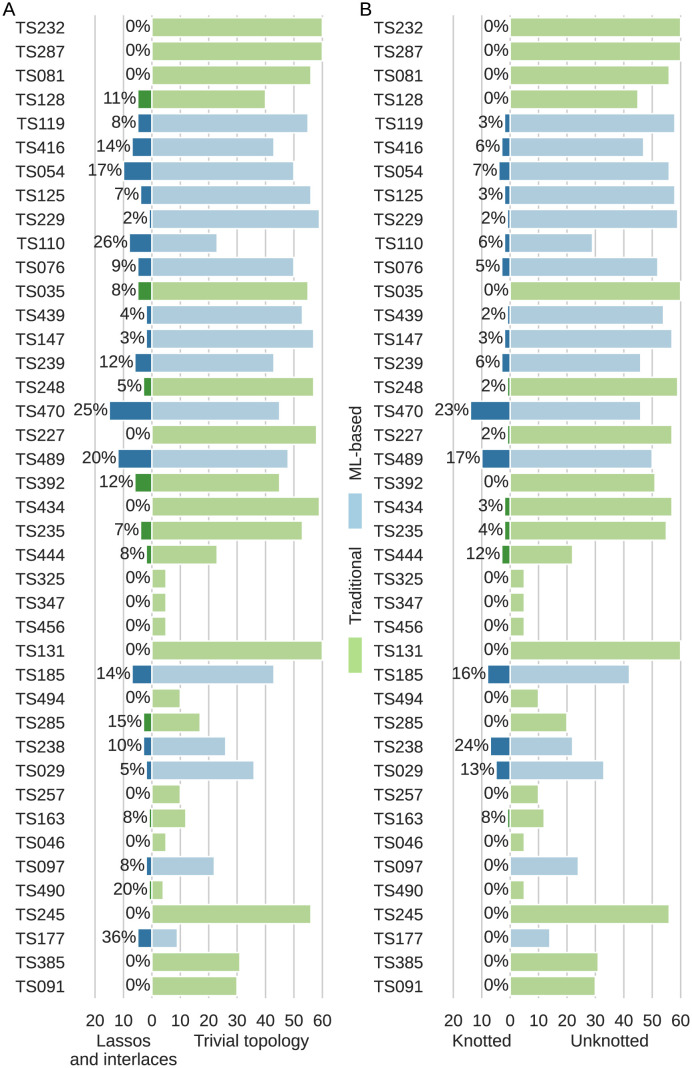
Distribution of entangled 3D RNA structure predictions by method. Modeling groups (classified as traditional or ML-based) are listed due to their ranking in CASP15 with the best one at the top. Results are shown separately for (A) entanglements of structure elements (lassos and interlaces) and (B) topological knots.

Next, we analyzed 3D RNA models by prediction group and examined the problem of structure entanglement from the point of view of the method. We checked how many nontrivial topologies, distinguishing between topological knots, lassos, and interlaces, appear in the models generated by a given prediction method (Fig 4). Of the 41 groups, 16 submitted only unentangled models (a total of 510 predictions, which makes 30% of all predictions). The remaining 25 groups predicted 1,150 models, of which 126 (10%) include entanglements of structure elements, and 78 (6%) are knotted from the topological point of view.

We divided the predictive methods into traditional and ML-based according to the information provided in the CASP15 book of abstracts [47]. Groups that applied machine learning predicted more models on average than groups using traditional methods—17/41 groups (41%) used ML and predicted 814/1,660 models (49%); 24/41 groups (59%) used traditional approaches and submitted 846/1,660 models (51%). Among 814 RNA models generated by machine learning-based methods, 132 included entangled structure elements, and 67 had a topological knot (34 were trefoils). For comparison, in the set of 846 RNA predictions by traditional algorithms, we found only 33 with entangled structure elements, and 10 with a knot (6 were trefoils). It follows that ML methods are 4x more prone to generate models containing entanglements of structural elements and 7x more prone to predict a knotted model than traditional algorithms. In particular, ML methods generated 6x more trefoils and 8x more complex knots than non-ML approaches. Moreover, all 20 structures, which were too tangled to check their knot type (TTC), were also predicted by ML methods. The distribution of the entanglement types is presented in Table 2.

**Table 2:** Entanglements in RNA predictions by method.

Predicted by	#Models	#Interlaces	#Lassos	#Trefoils	#Other knots	#TTC
traditional methods	846	62	103	6	4	0
ML-based methods	814	111	191	34	33	20
servers	423	47	66	7	10	8
human predictors	1,237	126	228	33	27	12
total number	1,660	173	294	40	37	20

Finally, recall that CASP accepts submissions from two categories of participants, web servers, and human groups. Predictions from the former category are fully automatic and must be submitted within 72 hours of publishing the target sequence. Human groups have 3 weeks to make predictions and can utilize any method to support the modeling process, including laboratory experiments to refine their models. With this in mind, we checked whether topological knots and entanglements of structure elements are more often in the web server than in human predictions. Among the 41 participants, there were 9 web servers. They submitted a total of 423 models, including 29 (0.07%) with entanglements. 32 human groups predicted 1,237 models, of which 97 (0.08%) were entangled. The number of entanglements of each type in the models submitted by both categories of participants is shown in Table 2. Based on these data, the thesis that automated predictions are more likely to get entangled than those submitted by experts cannot be confirmed.

### Example predictions with artifacts

**Fig 5:**
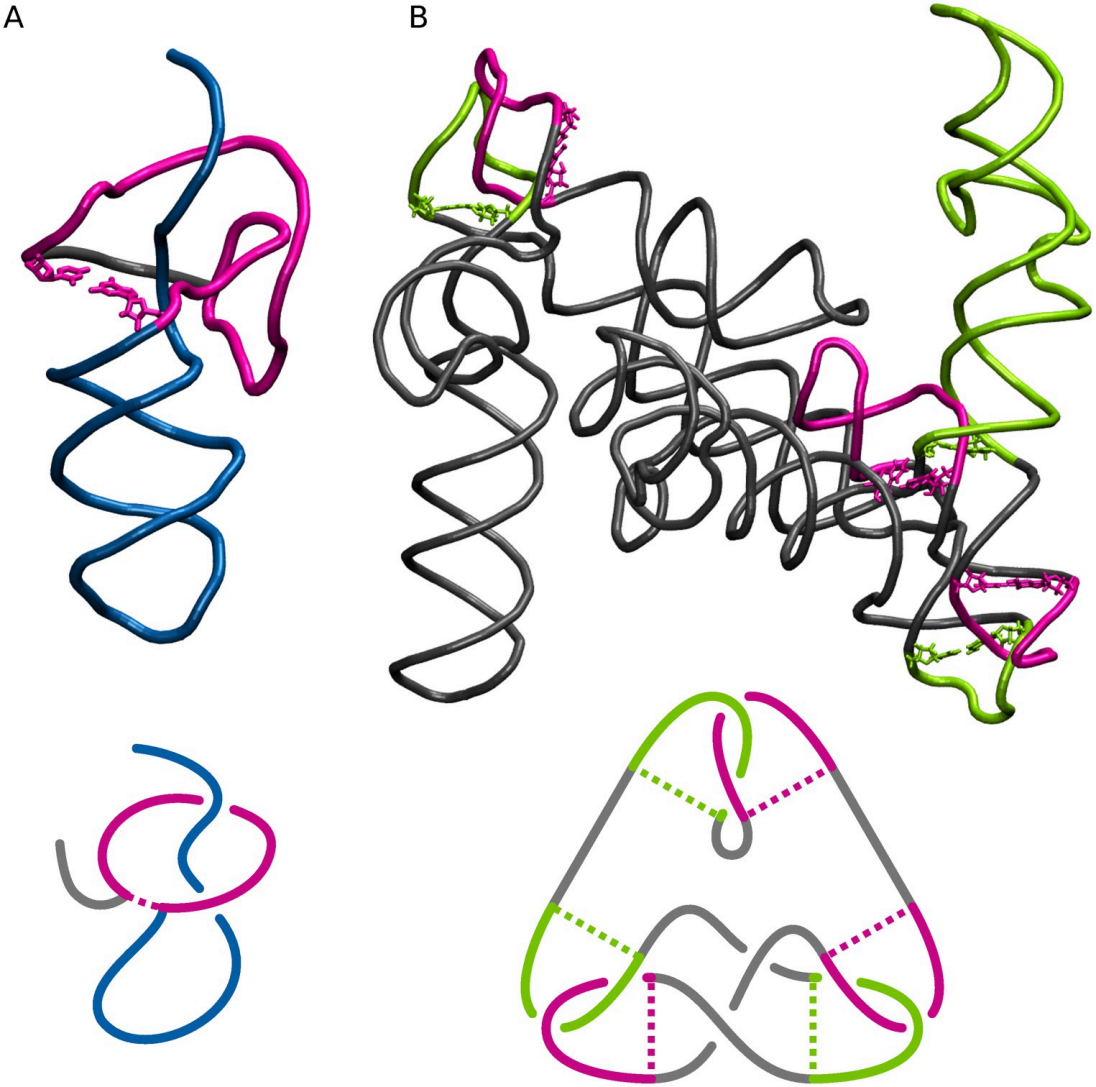
Two models with entangled structural elements predicted by ML-based methods and diagrams showing included entanglements. (A) The R1107TS416_2 model (69 nt) with a lasso. (B) The R1136TS110_4 model (375 nt) with highlighted three interlaces forming the 3_1_ knot. Hydrogen bonds closing an entangled loop are marked with dotted lines. Secondary structures of both models are shown in S2 Fig.

First, let us present the 3D RNA prediction that contains a lasso and was generated using the machine learning-based method. The R1107TS416_2 model was submitted by the TS416 group (AIchemy_RNA). It targeted the natural 69-nt-long RNA structure of the human CPEB3 HDV-like ribozyme (PDB ID: 7QR4) [48] (target ID: R1107). The native structure contains a pseudoknot formed between the dangling 5’-end (residues 1–6) and the three-way junction (residues 31–36). The R1107TS416_2 model is not an ideal reconstruction of the target either from the point of view of secondary structure (S2 Fig) (INF_*all*_ = 0.77) or the 3D topology (RMSD = 7.81Å, TM-score = 0.392). The model contains the L(S)-type lasso formed between the 26-nt-long hairpin closed by a pseudoknotted base pair 0:6–0:31 and a 34-nt-long single strand (0:36–0:69) (Fig 5A). Both of these structure elements also exist in the native structure, although in the latter a single strand bypasses the loop. In the predicted model, the intersection of the area inside the loop is between residues 0:A62 and 0:U63 of the puncture strand. The single strand passes quite a distance from the chain that forms the loop. Therefore, we did not observe clashed atoms in this part of the structure; in general, the Clash score is low and equals 14.02. According to the threshold adopted for shallow lassos, the entanglement found belongs to the deep category; its depth equals 6 nucleotides.

The other example, R1136TS110_4 model, was submitted by the TS110 group (DF_RNA). It is an ML-driven prediction of the synthetic construct, which is the 3D structure of a brocolli-pepper aptamer FRET tile in the ligand-bound state (PDB ID: 7ZJ4) [49] (target ID: R1136). The reference structure consists of 374 nucleotides and has a non-trivial topology with kissing-loop interactions between two hairpins (0:50-0:60+0:142-0:152; 0:75-0:85+0:121-0:131). The secondary structure of the predicted model (S2 Fig) is quite well reconstructed (INF_*all*_ = 0.82), while the 3D fold clearly deviates from the native (RMSD = 44.35Å, TM-score = 0.304). Clash score = 49.78. The model contains six entanglements of structural elements—two D(D), two D&D, one D&L, and one D(L) (Fig 5B), formed between two 10-nt-long hairpin loops (0:142-0:152; 0:50-0:60) and seven dinucleotide steps (0:119-0:120+0:132-0:133; 0:159-0:160+0:216-0:217; 0:160-0:161+0:215-0:216; 0:335-0:336+0:345-0:346; 0:49-0:50+0:60-0:61; 0:336-0:337+0:344-0:345; 0:293-0:294+0:299-0:300). They are not only artifacts of modeling but also incorrect conformations hardly possible to form while RNA folding.

**Fig 6:**
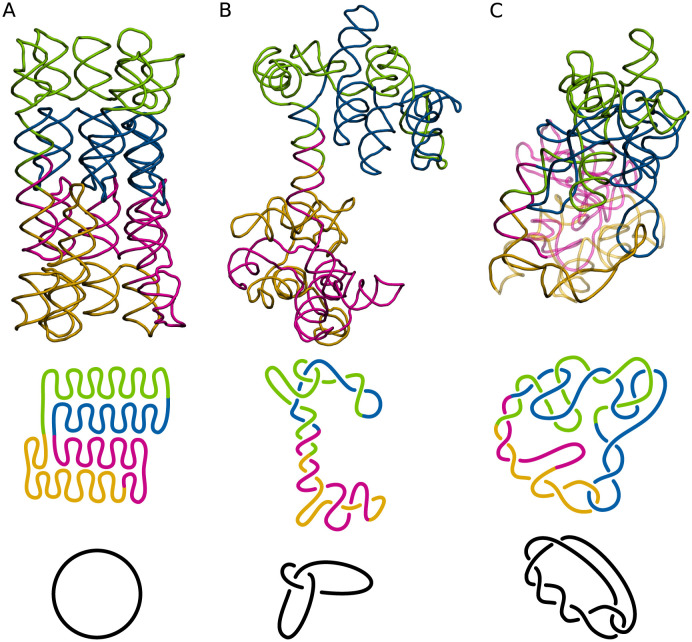
3D models predicted for the same target containing various topological knots and schematics of the latter. (A) Target structure R1138 (mature, 720 nt). (B) The R1138TS227_2 model generated by traditional method. (C) The R1138TS054_3 model predicted by ML-based method. Secondary structures of all three models are shown in S3 Fig.

The next example presents different topological knots in the 3D RNA models predicted for the largest RNA target of CASP15, that is, R1138 (720 nts). The native structure (synthetic construct) is a young conformer of a 6-helix bundle of RNA with clasp (PDB IDs: 7PTK, 7PTL) [50]. As shown in Fig 6A, it does not contain topological knots. Model R1138TS227_2 (Fig 6B) was generated by a traditional (non-ML) approach used by the TS227 group (GinobiFold). RMSD = 48.23Å and TM-score = 0.204 indicate a significant deviation of its 3D topology from the target structure, while the secondary structure is quite well-reproduced (INF_*all*_ = 0.82). Interestingly, clashes are almost non-existent (Clash score = 0.30). This model is knotted and forms a trefoil (3_1_), the simplest non-trivial knot. On the other hand, the R1138TS054_3 model was predicted using the machine learning-based method by the TS054 group (UltraFold). Its ratings are similar to the previous example (RMSD = 39.73Å, TM-score = 0.186, INF_*all*_ = 0.85, and Clash score = 4.81), however, this structure forms a more complicated 7_2_ knot. This clearly shows that existing evaluation measures are not correlated with the complexity of the structure entanglement.

## Conclusion

An analysis of the 1,660 3D RNA models predicted within CASP15 showed that predictive methods using machine learning are four times more likely than traditional tools to generate structures with entanglements, which are artifacts of the computational process. We hypothesize that the generation of entanglements may be due to the algorithm prioritization of more compact structures. The predictions submitted by web servers and human groups contain the same percentages of entangled models. The implication is that predictors do not use any, either automatic or non-automatic, verification of their models for topological anomalies. The characteristics of the modeled structure also appear to affect the probability of entanglement. In the set of all predictions, the largest number of entangled models was generated for large targets of synthetic RNA molecules. The probability of entanglement in this subset was significantly higher than for natural structures. We believe that enriching prediction methods with procedures to validate the topology would increase the accuracy of 3D RNA structure prediction. Currently, such validation can be easily done with the RNAspider and Topoly packages applied in the presented work. However, in the future, other ways of measuring topological defects could be designed, such as a loss function that would identify incorrect entanglements and fix them. This is not a straightforward task. Well-established methods for the recognition of knotted closed chain topologies are knot invariants such as Alexander, Jones, and HOMFLY-PT polynomials [44, 51–53]. All of them are computed using skein relations, which have exponential time complexity dependent on the number of crossings on a polymer projection. The exception is the in-point calculation of the Alexander polynomial that uses a determinant of the Alexander matrix and runs with a polynomial-time complexity. However, RNA structures are mainly open chains. For such cases, the Jones polynomial for knotoids [54–56] can be used, the value of which continuously changes with the coordinates of the chain. The alternative is to close the chain and use methods designed for closed chains [57]. Chain closure allows safe use of the KMT chain reduction algorithm [58] and greatly reduces the complexity of the chain to analyze. However, note that different chain closures can result in different knots. Due to the time of gradient computation, none of the above methods seems suitable to be applied as a loss function for predictive algorithms. More suitable candidates for a loss function might be machine learning models trained specifically for knot recognition [59–61].

## Supporting information

S1 Table Entangled RNA 3D models predicted in CASP15. (ODS)

S1 Fig Computational pipeline used to identify and analyze entangled 3D RNA models in CASP15 submissions. (PDF)

S2 Fig Secondary structures of two models with entangled structure elements predicted by ML-based methods. (A) The R1107TS416_2 model (69 nt) with a lasso. (B) The R1136TS110_4 model (375 nt) with two D&D entanglements (D1 interlaced with D1′ and D2 interlaced with D2′) and one D&L entanglement (D3 interlaced with L3). Colors match those applied in Fig 5.
(EPS)

S3 Fig Secondary structures of models predicted for the same target containing various topological knots. (A) Target structure R1138 (mature, 720 nt). (B) The R1138TS227_2 model generated by a traditional method. (C) The R1138TS054_3 model predicted by an ML-based method. Colors match those applied in Fig 6.
(EPS)

## References

[1] Pereira J, Simpkin AJ, Hartmann MD, Rigden DJ, Keegan RM, Lupas AN. High-accuracy protein structure prediction in CASP14. Proteins. 2021;89(12):1687–1699. doi: 10.1002/prot.26171 34218458

[2] Schneider B, Sweeney B, Bateman A, Cerny J, Zok T, Szachniuk M. When will RNA get its AlphaFold moment? Nucleic Acids Res. 2023;51(18):9522–9532. doi: 10.1093/nar/gkad726 37702120 PMC10570031

[3] Parisien M, Cruz JA, Westhof E, Major F. New metrics for comparing and assessing discrepancies between RNA 3D structures and models. RNA. 2009;15(10):1875–1885. doi: 10.1261/rna.1700409 19710185 PMC2743038

[4] Szachniuk M. RNApolis: computational platform for RNA structure analysis. Foundations of Computing and Decision Sciences. 2019;44(2):241–257. doi: 10.2478/fcds-2019-0012

[5] Wiedemann J, Zok T, Milostan M, Szachniuk M. LCS-TA to identify similar fragments in RNA 3D structures. BMC Bioinform. 2017;18:456. doi: 10.1186/s12859-017-1867-6 29058576 PMC5651598

[6] Magnus M, Antczak M, Zok T, Wiedemann J, Lukasiak P, Cao Y, et al. RNA-Puzzles toolkit: A computational resource of RNA 3D structure benchmark datasets, structure manipulation, and evaluation tools. Nucleic Acids Res. 2020;48(2):576–588. doi: 10.1093/nar/gkz1108 31799609 PMC7145511

[7] Carrascoza F, Antczak M, Miao Z, Westhof E, Szachniuk M. Evaluation of the stereochemical quality of predicted RNA 3D models in the RNA-Puzzles submissions. RNA. 2021;28(2):250–262. doi: 10.1261/rna.078685.121 34819324 PMC8906551

[8] Das R, Kretsch RC, Simpkin AJ, Mulvaney T, Pham P, Rangan R, et al. Assessment of three-dimensional RNA structure prediction in CASP15. Proteins: Struct, Funct, Bioinf. 2023;91(12):1747–1770. doi: 10.1002/prot.26602 PMC10841292 37876231

[9] Popenda M, Zok T, Sarzynska J, Korpeta A, Adamiak RW, Antczak M, et al. Entanglements of structure elements revealed in RNA 3D models. Nucleic Acids Res. 2021;49(17):9625–9632. doi: 10.1093/nar/gkab716 34432024 PMC8464073

[10] Zając S, Geary C, Andersen ES, Dabrowski-Tumanski P, Sulkowska JI, Sułkowski P. Genus trace reveals the topological complexity and domain structure of biomolecules. Sci Rep. 2018;8(1):17537. doi: 10.1038/s41598-018-35557-3 30510290 PMC6277428

[11] Luwanski K, Hlushchenko V, Popenda M, Zok T, Sarzynska J, Martsich D, et al. RNAspider: a webserver to analyze entanglements in RNA 3D structures. Nucleic Acids Res. 2022;50(W1):W663–W669. doi: 10.1093/nar/gkac218 35349710 PMC9252836

[12] VanLoock M, Harris B, Harvey S. To knot or not to knot? Examination of 16s ribosomal RNA models. J Biomol Struct Dyn. 1998;16(3):709–713. doi: 10.1080/07391102.1998.10508282 10052626

[13] Poblete S, Guzman HV. Structural 3D domain reconstruction of the RNA genome from viruses with secondary structure models. Viruses. 2021;13(8):1555. doi: 10.3390/v13081555 34452420 PMC8402887

[14] Cruz-León S, Assenza S, Poblete S, Guzman HV. In: Comas-Garcia M, Rosales-Mendoza S, editors. RNA multiscale simulations as an interplay of electrostatic, mechanical properties, and structures inside viruses. Springer International Publishing; 2023. p. 27–56.

[15] Micheletti C, Di Stefano M, Orland H. Absence of knots in known RNA structures. PNAS. 2015;112(7):2052–2057. doi: 10.1073/pnas.1418445112 25646433 PMC4343165

[16] Burton AS, Di Stefano M, Lehman N, Orland H, Micheletti C. The elusive quest for RNA knots. RNA Biol. 2016;13(2):134–139. doi: 10.1080/15476286.2015.1132069 26828280 PMC4829277

[17] Niemyska W, Mukherjee S, Gren B, Niewieczerzal S, Bujnicki JM, Sulkowska JI. Discovery of a trefoil knot in the RydC RNA: challenging previous notions of RNA topology. J Mol Biol. 2024;436(6):168455. doi: 10.1016/j.jmb.2024.168455 38272438

[18] Dimastrogiovanni D, Frohlich KS, Bandyra KJ, Bruce HA, Hohensee S, Vogel J, et al. Recognition of the small regulatory RNA RydC by the bacterial Hfq protein. eLife. 2014;3:e05375. doi: 10.7554/eLife.05375 25551292 PMC4337610

[19] Finci LI, Zhang X, Huang X, Zhou Q, Tsai J, Teng T, et al. The cryo-EM structure of the SF3b spliceosome complex bound to a splicing modulator reveals a pre-mRNA substrate competitive mechanism of action. Genes Dev. 2018;32:309–320. doi: 10.1101/gad.311043.117 29491137 PMC5859971

[20] King NP, Yeates EO, Yeates TO. Identification of rare slipknots in proteins and their implications for stability and folding. J Mol Biol. 2007;373(1):153–166. doi: 10.1016/j.jmb.2007.07.042 17764691

[21] Sulkowska JI, Rawdon EJ, Millet KC, Onuchic JN, Stasiak A. Conservation of complex knotting and slipknotting patterns in proteins. Biophys J. 2012;102(3):253a. doi: 10.1073/pnas.1205918109 22685208 PMC3387036

[22] Hou YM, Matsubara R, Takase R, Masuda I, Sulkowska JI. TrmD: a methyl transferase for tRNA methylation with m1G37. The Enzymes. 2017;41:89–115. doi: 10.1016/bs.enz.2017.03.003 28601227 PMC6054489

[23] Sulkowska JI. On folding of entangled proteins: knots, lassos, links and *θ*-curves. Curr Opin Struct Biol. 2020;60:131–141. doi: 10.1016/j.sbi.2020.01.007 32062143

[24] Hsu STD. Folding and functions of knotted proteins. Curr Opin Struct Biol. 2023;83:102709. doi: 10.1016/j.sbi.2023.102709 37778185

[25] Jamroz M, Niemyska W, Rawdon EJ, Stasiak A, Millett KC, Sułkowski P, et al. KnotProt: a database of proteins with knots and slipknots. Nucleic Acids Res. 2015;43(D1):D306–D314. doi: 10.1093/nar/gku1059 25361973 PMC4383900

[26] da Silva FB, Lewandowska I, Kluza A, Niewieczerzal S, Augustyniak R, Sulkowska JI. First crystal structure of double knotted protein TrmD-Tm1570–inside from degradation perspective. bioRxiv [pre-print]. 2023; p. 2023–03.

[27] Hsu MF, Sriramoju MK, Lai CH, Chen YR, Huang JS, Ko TP, et al. Structure, dynamics, and stability of the smallest and most complex 71 protein knot. J Biol Chem. 2024;300(1). doi: 10.1016/j.jbc.2023.105553 38072060 PMC10840475

[28] Niemyska W, Rubach P, Gren BA, Nguyen ML, Garstka W, Bruno da Silva F, et al. AlphaKnot: server to analyze entanglement in structures predicted by AlphaFold methods. Nucleic Acids Res. 2022;50(W1):W44–W50. doi: 10.1093/nar/gkac388 35609987 PMC9252816

[29] Sumners D, Whittington S. Knots in self-avoiding walks. J Phys A Math Gen. 1988;21(7):1689. doi: 10.1088/0305-4470/21/7/030

[30] Pippenger N. Knots in random walks. Discrete Appl Math. 1989;25(3):273–278. doi: 10.1016/0166-218X(89)90005-X

[31] Deguchi T, Tsurusaki K. Universality of random knotting. Phys Rev E. 1997;55(5):6245. doi: 10.1103/PhysRevE.55.6245

[32] Sulkowska JI, Sulkowski P, Onuchic J. Dodging the crisis of folding proteins with knots. Biophys J. 2009;96(3):81a. doi: 10.1073/pnas.0811147106 19211785 PMC2651233

[33] Li W, Terakawa T, Wang W, Takada S. Energy landscape and multiroute folding of topologically complex proteins adenylate kinase and 2ouf-knot. PNAS. 2012;109(44):17789–17794. doi: 10.1073/pnas.1201807109 22753508 PMC3497823

[34] Sułkowska JI, Noel JK, Onuchic JN. Energy landscape of knotted protein folding. PNAS. 2012;109(44):17783–17788. doi: 10.1073/pnas.1201804109 22891304 PMC3497829

[35] a Beccara S, Škrbić T, Covino R, Micheletti C, Faccioli P. Folding pathways of a knotted protein with a realistic atomistic force field. PLoS Comput Biol. 2013;9(3):e1003002. doi: 10.1371/journal.pcbi.1003002 23555232 PMC3605060

[36] Soler MA, Rey A, Faísca PF. Steric confinement and enhanced local flexibility assist knotting in simple models of protein folding. Phys Chem Chem Phys. 2016;18(38):26391–26403. doi: 10.1039/C6CP05086G 27722468

[37] Jackson SE, Suma A, Micheletti C. How to fold intricately: using theory and experiments to unravel the properties of knotted proteins. Curr Opin Struct Biol. 2017;42:6–14. doi: 10.1016/j.sbi.2016.10.002 27794211

[38] Dabrowski-Tumanski P, Sulkowska JI. Topological knots and links in proteins. PNAS. 2017;114(13):3415–3420. doi: 10.1073/pnas.1615862114 28280100 PMC5380043

[39] Dabrowski-Tumanski P, Stasiak A. AlphaFold Blindness to Topological Barriers Affects Its Ability to Correctly Predict Proteins’ Topology. Molecules. 2023;28(22):7462. doi: 10.3390/molecules28227462 38005184 PMC10672856

[40] Sramkova D, Sikora M, Uchal D, Klimentova E, Perlinska AP, Nguyen ML, et al. Knot or Not? Sequence-Based Identification of Knotted Proteins With Machine Learning. bioRxiv [pre-print]. 2023; p. 2023–09.

[41] Dabrowski-Tumanski P, Rubach P, Niemyska W, Gren BA, Sulkowska JI. Topoly: Python package to analyze topology of polymers. Brief Bioinform. 2021;22(3):bbaa196. doi: 10.1093/bib/bbaa196 32935829 PMC8138882

[42] Kryshtafovych A, Antczak M, Szachniuk M, Zok T, Kretsch RC, Rangan R, et al. New prediction categories in CASP15. Proteins: Struct, Funct, Bioinf. 2023;91(12):1550–1557. doi: 10.1002/prot.26515 PMC10713864 37306011

[43] Zok T. BioCommons: A Robust Java Library for RNA Structural Bioinformatics. Bioinformatics. 2021;37(17):2766–2767. doi: 10.1093/bioinformatics/btab069 33532837 PMC8428578

[44] Alexander JW. Topological invariants of knots and links. Trans Am Math Soc. 1928;30(2):275–306. doi: 10.1090/S0002-9947-1928-1501429-1

[45] Millett KC, Rawdon EJ, Stasiak A, Sulkowska JI. Identifying knots in proteins. Biochem Soc Trans. 2013;41(2):533–537. doi: 10.1042/BST20120339 23514149

[46] Jamroz M, Niemyska W, Rawdon EJ, Stasiak A, Millett KC, Sułkowski P, et al. KnotProt: a database of proteins with knots and slipknots. Nucleic Acids Res. 2015;43(D1):D306–D314. doi: 10.1093/nar/gku1059 25361973 PMC4383900

[47] CASP15 Abstract book; 2022. Available from: https://predictioncenter.org/casp15/doc/CASP15_Abstracts.pdf.

[48] Przytula-Mally AI, Engilberge S, Johannsen S, Olieric V, Masquida B, Sigel RK. Anticodon-like loop-mediated dimerization in the crystal structures of HdV-like CPEB3 ribozymes. bioRxiv [pre-print]. 2022; p. 2022–2209.

[49] Kretsch RC, Andersen ES, Bujnicki JM, Chiu W, Das R, Luo B, et al. RNA target highlights in CASP15: Evaluation of predicted models by structure providers. Proteins: Struct, Funct, Bioinf. 2023;91(12):1600–1615. doi: 10.1002/prot.26550 37466021 PMC10792523

[50] McRae EKS, Rasmussen HO, Liu J, Boggild A, Nguyen MTA, Sampedro Vallina N, et al. Structure, folding and flexibility of co-transcriptional RNA origami. Nat Nanotechnol. 2023;18:808–817. doi: 10.1038/s41565-023-01321-6 36849548 PMC10566746

[51] Jones VF. Hecke algebra representations of braid groups and link polynomials. In: New Developments In The Theory Of Knots. World Scientific; 1987. p. 20–73.

[52] Freyd P, Yetter D, Hoste J, Lickorish WR, Millett K, Ocneanu A. A new polynomial invariant of knots and links. Bull Amer Math Soc. 1985;12(2):239–246. doi: 10.1090/S0273-0979-1985-15361-3

[53] Przytycki JH, Traczyk P. Invariants of links of Conway type. arXiv [pre-print] arXiv:161006679. 2016;.

[54] Gügümcü N, Kauffman LH. New invariants of knotoids. Eur J Comb. 2017;65:186–229. doi: 10.1016/j.ejc.2017.06.004

[55] Dorier J, Goundaroulis D, Benedetti F, Stasiak A. Knoto-ID: a tool to study the entanglement of open protein chains using the concept of knotoids. Bioinformatics. 2018;34(19):3402–3404. doi: 10.1093/bioinformatics/bty365 29722808

[56] Panagiotou E, Kauffman LH. Knot polynomials of open and closed curves. P Roy Soc A. 2020;476(2240):20200124. doi: 10.1098/rspa.2020.0124 32922152 PMC7482204

[57] Millett KC, Rawdon EJ, Stasiak A, Sułkowska JI. Identifying knots in proteins. Biochem Soc Trans. 2013;41(2):533–537. doi: 10.1042/BST20120339 23514149

[58] Koniaris K, Muthukumar M. Self-entanglement in ring polymers. J Chem Phys. 1991;95(4):2873–2881. doi: 10.1063/1.460889

[59] Vandans O, Yang K, Wu Z, Dai L. Identifying knot types of polymer conformations by machine learning. Phys Rev E. 2020;101(2):022502. doi: 10.1103/PhysRevE.101.022502 32168694

[60] Gukov S, Halverson J, Ruehle F, Sułkowski P. Learning to unknot. Mach Learn Sci Technol. 2021;2(2):025035. doi: 10.1088/2632-2153/abe91f

[61] Bruno da Silva F, Gabrovsek B, Korpacz M, Luczkiewicz K, Niewieczerzal S, Sikora M, et al. Knots and *θ*-Curves Identification in Polymeric Chains and Native Proteins Using Neural Networks. Macromolecules. 2024;. doi: 10.1021/acs.macromol.3c02479

