# Supplementary figures and images for: Knotted artifacts in predicted 3D RNA structures
Source: PLoS Comput Biol. 2024 Jun 20;20(6):e1011959. doi: 10.1371/journal.pcbi.1011959 (PMC11218946; doi:10.1371/journal.pcbi.1011959)

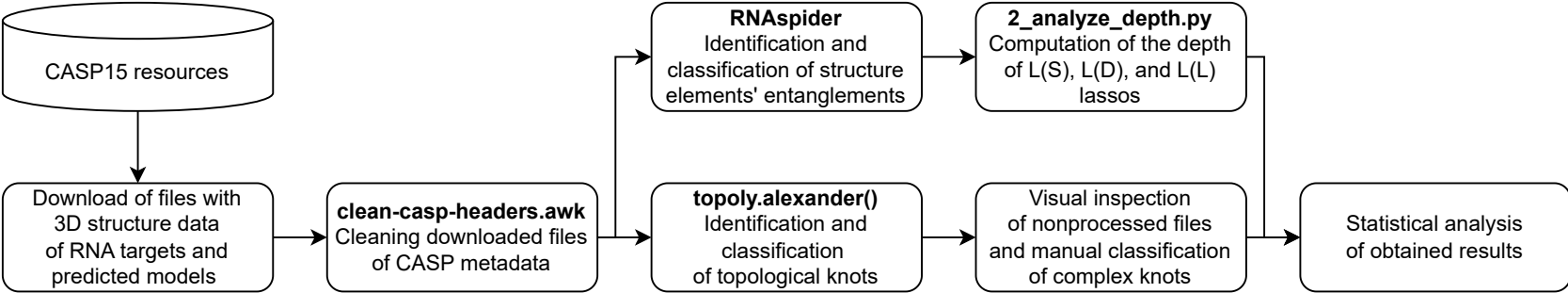

Supplement: S1 Fig — (PDF) [file pcbi.1011959.s002.pdf]
